# The maturation of iPS cell-derived brain microvascular endothelial cells by inducible-SOX18 expression

**DOI:** 10.1186/s12987-023-00408-5

**Published:** 2023-02-02

**Authors:** Hongyan Zhang, Tomoko Yamaguchi, Kenji Kawabata

**Affiliations:** 1 Laboratory of Biomedical Innovation, Graduate School of Pharmaceutical Sciences, Osaka University, 1-6 Yamadaoka, Suita, Osaka 565-0871 Japan; 2 Laboratory of Cell Model for Drug Discovery, National Institutes of Biomedical Innovation, Health, and Nutrition, Saito-Asagi 7-6-8, Ibaraki, Osaka 567-0085 Japan

**Keywords:** Blood-brain-barrier, Brain microvascular endothelial cells, Sox18, Transcription factors, Maturation, Differentiation

## Abstract

**Background:**

Brain microvascular endothelial cells (BMECs) play a major role in the blood–brain barrier (BBB), and are critical for establishing an in vitro BBB model. Currently, iPSC-derived BMECs (iBMECs) have been used to construct in vitro BBB models with physiological barrier functions, such as high trans-endothelial electrical resistance (TEER) and expression of transporter proteins. However, the relatively low p-glycoprotein (P-gp) level and a decrease in the efflux ratio of its substrates in iBMECs suggest their immature nature. Therefore, more mature iBMECs by optimizing the differentiation induction protocol is beneficial for establishing a more reliable in vitro BBB model for studying central nervous system (CNS) drug transport.

**Methods:**

To identify human brain endothelial cell fate-inducing factors, HUVEC was transfected with Zic3A-, Zic3B-, and Sox18-expressing lentivirus vector. Since SOX18 was found to induce BMEC properties, we used a Dox-inducible Tet-on system to express SOX18 during iBMEC differentiation and explored the impact of SOX18 expression on iBMEC maturation.

**Results:**

Sox18-mediated iBMECs achieved a higher TEER value than normal iBMECs (> 3000 Ω cm^2^). From day 6 to day 10 (d6–10 group), the iBMECs with SOX18 expression expressed a series of tight junction markers and showed upregulation of Mfsd2a, a specific marker of the BBB. The d6–10 group also expressed SLC2A1/Glut1 at levels as high as normal iBMECs, and upregulated ABCB1/P-gp and ABCC1/MRP1 expression. Moreover, Sox18-mediated iBMECs showed higher viability than normal iBMECs after puromycin treatment, indicating that SOX18 expression could upregulate P-gp activity in iBMECs.

**Conclusions:**

Inducible SOX18 expression in iBMECs gained BBB phenotypes, including high TEER values and upregulation of tight junction-related genes, endothelial cell (EC) markers, BBB transporters, and higher cell viability after treatment with puromycin. Collectively, we provide a differentiation method for the maturation of human iPS cell-derived BMECs with SOX18 expression, describing its contribution to form an in vitro BBB model for CNS drug transport studies.

**Supplementary Information:**

The online version contains supplementary material available at 10.1186/s12987-023-00408-5.

## Background

The blood-brain barrier (BBB) is a unique property of the central nervous system (CNS) microvasculature that regulates CNS homeostasis and protects the brain by limiting the movement of ions, minerals, and cells between blood and CNS. Brain microvascular endothelial cells (BMECs) connected by tight junctions help in the passive diffusion of molecules across the brain, causing an extremely high trans-endothelial electrical resistance (TEER) in the blood vessels. In addition, BMECs expresses a series of specialized efflux transporters that regulate the transport of substances, such as p-glycoprotein (P-gp), to maintain brain homeostasis.

Initially, BMECs derived from primary and immortalized cell lines from mice, rats, bovines, and humans were used for developing in vitro BBB model. However, many reasons, such as differences between species in transporter expression patterns, loss of BBB function during in vitro culture, and time and monetary costs, limit the development of these models. Recently, human-induced pluripotent stem cell (iPSC)-derived BMECs (iBMECs) have been used in BBB models, exhibiting normal barrier function and expression of specific transporters [1]. Using a co-differentiation strategy, Lippmann et al. differentiated iPSCs into neural and endothelial lineage cells in an unconditioned medium. The unique properties of BBB, including high TEER values, normal tube formation, expression of specific transporters, and LDL uptake was acquired by treating the cells with retinoic acid [2, 3]. Even though the method has been used widely by many researchers, there is still room for improvement in iBMECs developed using a co-differentiation strategy [1, 4]. A genome-wide expression profiling exploring the transcriptional changes in iBMECs showed that iBMECs obtained by the co-differentiation protocol had lower levels of vascular endothelial (VE)-cadherin and claudin-5 (CLDN5) but had higher levels of CLDN7 and others, presenting both endothelial and epithelial features[4]. Moreover, multiple studies have reported that iBMECs express P-gp at a relatively low level, with a decreased efflux ratio of its substrates, suggesting immature nature of iBMECs [1, 4]. These results suggest that further characterization of iBMECs is essential for developing reliable in vitro BBB models.

SRY-box transcription factor 18 (Sox18) encodes a member of the Sry-related high-mobility group box (SOX) family transcription factors (TFs). Sox18 is critical for angiogenesis during wound healing and tissue repair and plays a vital role in controlling endothelial barrier formation [5, 6]. Moreover, Sox18 is a barrier-inducing TF in endothelial cells (ECs) that can upregulate Wnt-related signaling and downregulate EC proliferation [7]. In addition to Sox18, the zinc finger protein of the cerebellum (Zic) family member, Zic3, is also highly enriched in BMECs, which plays a prominent role in both canonical Wnt signal activation and Zic3 DNA-binding activity in BMEC-specific gene expression [8].

To identify TFs that are effective in inducing BBB phenotypes, Zic3A, Zic3B, and Sox18 were transfected into human umbilical vein endothelial cells (HUVECs). The expression of SOX18 induced the upregulation of EC and BBB markers in HUVECs, implying that Sox18 has the potential to promote iBMEC maturation. We then differentiated BMECs from iPSC using inducible SOX18 expression to induce iBMEC maturation.

## Methods

### Cell culture

The human iPS cell line iMR90-4 [9] in normal group and transfected groups were cultured in a modified Tenneille Serum Replacer 1 (mTeSR1) medium (Stem Cell Technologies), with daily medium replacement. Cells were passaged with a growth factor-reduced BD Matrigel Basement Membrane Matrix (Corning) to form a confluent monolayer culture, including TrypLE Select enzyme (ThermoFisher Scientific). HUVECs were cultured in a basal medium (EGM-2, Lonza).

### Lentivirus production

pcDNA3.1(-)-hSox18 (Gene Script) was subcloned into the NotI/BamHI sites of the CSII-EF-MCS-IRES2-Venus lentivirus vector (RIKEN BioResource Center) to create CSII-EF-Sox18-IRES2-Venus. Zic3A/B was cloned into the EcoRV site of pcDNA, yielding pcDNA-Zic3A/B, which was subcloned into the NotI/BamHI sites of the CSII-EF-MCS-IRES2-Venus generating CSII-EF-Zic3A/B-IRES2-Venus.

For inducible Sox18 (iSox18) expression, the EF-1α promoter was inserted into the AgeI site of CSIV-TRE-RfA-CMV-KT (RIKEN BioResource Center), creating CSIV-TRE-RfA-EF-KT, which included a tetracycline-responsive element promoter (TRE). pENTR1A-Sox18 was constructed by subcloning pcDNA3.1(-)-hSox18 into the pENTR1A vector (ThermoFisher Scientific) using the NotI/BamHI sites. The LR recombination reaction between the entry plasmid pENTR1A-Sox18 and gateway destination plasmid CSIV-TRE-RfA-EF-KT was performed to generate the CSIV-TRE-Sox18-EF-KT (iSox18 lentiviral vector) using the Gateway LR Clonase Plus enzyme mix (ThermoFisher Scientific).

To generate lentiviruses, 293 T cells were transfected with vectors (CSII-EF-MCS-IRES2-Venus, CSII-EF-Sox18-IRES2-Venus, CSII-EF-Zic3A-IRES2-Venus, CSII-EF-Zic3B-IRES2-Venus, or CSIV-TRE-EF-Sox18-KT) with packaging plasmids (pCMV-VSV-G-RSV-Rev and pCAG-HIVgp) using Lipofectamine 2000 (ThermoFisher Scientific). After 24 h, the culture medium was replaced with a fresh medium, and the medium containing lentivirus was collected by a 0.45-μm filter after 48 h and concentrated by centrifugation. Lentivirus particles were suspended in Hanks' Balanced Salt Solution (HBSS, NACALAI TESQUE).

### Lentivirus transduction

HUVECs were transduced with CSII-EF-MCS-IRES2-Venus (multiplicity of infection (MOI): 10), CSII-EF-Zic3A/B-IRES2-Venus (MOI: 10), and CSII-EF-Sox18-IRES2-Venus (MOI: 10), and the medium was changed the next day. Three days after transduction, cells were collected for the assay.

### Generation of doxycycline (Dox)-inducible SOX18-expressing iPS cells

The iMR90-4 cells were transduced with the iSox18 lentiviral vector, dissociated using Accutase (Merk-Millipore), and resuspended in mTeSR1 supplemented with 10 μM Y27632 (Wako). Kusabira orange (KO)-positive cells were purified using an SH800 cell sorter (SONY) and seeded onto the growth factor-reduced BD Matrigel Basement Membrane Matrix in mTeSR1 medium with Y27632. Twenty-four individual colonies were selected and reseeded onto the Matrigel Basement Membrane Matrix 5 days post-sorting. KO-positive cells were identified using flow cytometry after the cells were cultured to near confluence. SOX18-mediated differentiation of iBMECs was conducted in Dox-inducible SOX18-iPS cells (iSox18-iPSCs) clone #6 and #20, and the results of clone #6 are described here. iSox18-iPSCs were cultured in a mTeSR1 medium, with daily medium replacement. Cells were passaged with a growth factor-reduced BD Matrigel Basement Membrane Matrix to form a confluent monolayer culture, including TrypLE Select enzyme.

### Generation of BMECs from iPS cells

iBMECs were differentiated from iMR90-4 cells according to a previously described protocol [3]. For SOX18-expressing cells, iMR90-4 cells transduced with the SOX18 expressing Tet-on system were expanded on Matrigel-coated plates in a defined mTeSR1 medium for 2–3 days. The cells were grown in an unconditioned medium for 6 days to initiate neural and endothelial co-differentiation. The iBMECs were further expanded for 4 days in an EC medium containing basic fibroblast growth factor (bFGF), all-trans retinoic acid (ATRA), and human platelet poor plasma derived serum (PDS; Sigma). As previously described by Lippmann et al., populations of BMECs selectively proliferate and acquire BBB phenotypes in the EC medium [3]. Thus, we used a Dox-inducible system to modulate SOX18 expression during the EC medium treatment period to observe the effects of SOX18 expression on iBMEC maturation. To induce the expression of SOX18, the cells were treated with Dox from day6 to day8 (d6–8), day8–day10 (d8–10), and day6 to day10 (d6–10). In this study, we defined SOX18-mediated iBMECs that were cultured without Dox as the “Normal group”.

### Reverse transcription and quantitative polymerase chain reaction (qRT-PCR)

A SuperScript VILO cDNA Synthesis Kit was used to generate first-strand cDNA. The resulting RNA was isolated using RNAiso Plus (Takara). The mRNA levels of Sox18, Zic3, ABCB1/P-gp, VE-cadherin, platelet endothelial cell adhesion molecule (PECAM1), plasmalemma vesicle-associated protein (Plvap), occludin (OCLN), tight junction protein 1 (TJP1)/ZO-1, CLDN5, major facilitator superfamily domain containing 2A (Mfsd2a), Solute Carrier Family 2 Member 1 (SLC2A1)/Glut1, ATP Binding Cassette Subfamily C Member 1 (ABCC1)/MRP1, ATP Binding Cassette Subfamily G Member 2 (ABCG2)/BCRP, Nanog, and octamer-binding transcription factor (Oct3/4) were determined using the SYBR Green detection system. Quantitative gene expression data were normalized to the housekeeping gene, glyceraldehyde 3-phosphate dehydrogenase (GAPDH). Primer sequences used in this study are listed in Table 1.

**Table 1:** Primer list used in quantitative real-time PCR

Gene Name	(5') Forward Primers (3')	(5') Reverse Primers (3')
GAPDH	GGTGGTCTCCTCTGACTTCAACGT	GTGGTCGTTGAGGGCAATG
Sox18	TTCCATGTCACAGCCCCCTAG	GACACGTGGGAACTCCAG
Zic3A	CAGCGACCGTAAGAAGCACA	ATAGCGGGTGGAGTGGAAGA
Zic3B	TGTGCATACCTCGGACAAGC	GTTATGGAGGGTGGCTGCT
ABCB1/P-gp	TTGTTTGCCACCACGATAGC	TTCTGCCCACCACTCAACTG
VE-cadherin	TCACGATAACACGGCCAACA	TGGCATCCCATTGTCTGAGA
PECAM1	GAGTATTACTGCACAGCCTTCA	AACCACTGCAATAAGTCCTTTC
Plvap	GCAGATGTGGCTGAATGCTC	CGCAGCTCTTGTTCATGTCC
OCLN	CAGCAGCGGTGGTAACTTTG	TCCCTGATCCAGTCCTCCTC
TJP1/ZO-1	TGATCATTCCAGGCACTCG	CTCTTCATCTCTACTCCGGAGACT
CLDN5	GTTCGTTGCGCTCTTCGTGA	GCTCGTACTTCTGCGACACG
Mfsd2a	CCCTATGAAGCCCAGCAGTC	TAAAGTGGCCGAGAGCATGA
SLC2A1/Glut1	GCTATGGGGAGAGCATCCTG	AAGGCCAGCAGGTTCATCAT
ABCC1/MRP1	CAAGGTGGATGCGAATGAGG	TGAGGAAGTAGGGCCCAAAG
ABCG2/BCRP	CTCTTCGGCTTGCAACAACT	TTCTCCTCCAGACACACCAC
Nanog	AGAAGGCCTCAGCACCTAC	GGCCTGATTGTTCCAGGATT
Oct3/4	CTTGAATCCCGAATGGAAAGGG	GTGTATATCCCAGGGTGATCCTC

### Measurement of TEER and NaF permeability

BBB barrier function was evaluated by measuring TEER. The resistance values (Ω) were measured using a Millicell ERS-2 (Merk Millipore). The TEER values were calculated as previously described [10, 11]. We confirmed the value of TEER was peaked on day 10. Therefore, all experiments were performed on day 10.

The cells were washed with Dulbecco’s phosphate-buffered saline (DPBS, Sigma) supplemented with D-glucose and HEPES (DPBS-H). DPBS-H containing 10 μg/mL sodium fluorescein (NaF, Sigma) as a paracellular marker was then added into the upper chamber for 30 min at 37 °C. After incubation, the medium in the lower chamber was collected and then the concentration of NaF in the medium was measured using a fluorescence multi-well plate reader (Genios, TECAN).

### Accumulation assay

The cells were incubated with 10 μM rhodamine 123 (Sigma) in transport buffer (distilled water with 0.12 M NaCl, 25 mM NaHCO_3_, 3 mM KCl, 2 mM MgSO_4_, 2 mM CaCl_2_, 0.4 mM K_2_HPO_4_, 1 mM HEPES, and 0.1% BSA) for 2 h at 37 °C. For inhibition experiments, cells were pre-incubated with 10 μM cyclosporin A (CsA; Sigma) for 60 min at 37 °C. Then, cells were washed three times with PBS and lysed with RIPA buffer. Fluorescence was measured using a fluorescence multi-well plate reader (Genios). Fluorescence was normalized on a per cell basis by counting dissociated cells.

### Cell viability assay

The cells were exposed to 1 μM puromycin (NACALAI TESQUE) for 48 h. The alamar Blue® cell viability reagent (10 ×) (Thermo Fisher Scientific) was directly added to the medium at 1/10th of the total volume. The cells were then incubated at 37 °C for 2 h. The absorbance was measured using an absorbance microplate reader (Sunrise, TECAN).

### Statistical analysis

One-way ANOVA followed by Tukey’s post hoc test was used for comparisons between more than two groups. Unpaired parametric two-tailed t tests were used for comparisons between two groups.

## Results

### Determination of transcription factor that can induce BMEC phenotype

**Fig.1:**
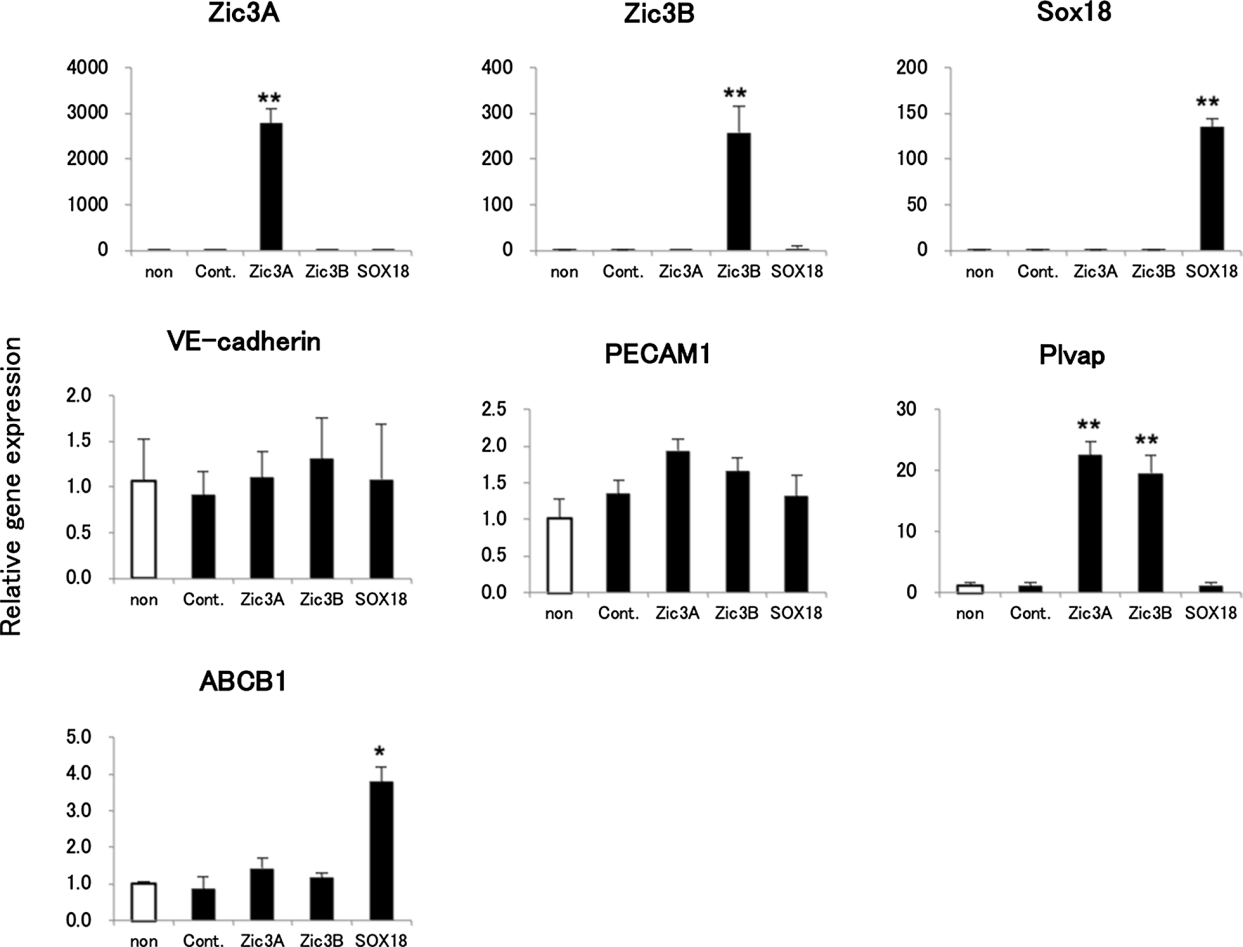
Brain endothelial fate-induced by transcription factors. Reverse transcription and quantitative polymerase chain reaction (qRT-PCR) analysis of purified RNA from HUVECs (non-transduced), CSII-EF-MCS-IRES-Venus (Control), CSII-EF-Zic3A-IRES-Venus (Zic3A), CSII-EF-Zic3B-IRES-Venus (Zic3B), and CSII-EF-Sox18-IRES-Venus (Sox18) transduced HUVECs. In the cells of the non-transduced group, the expression level of the gene was set to 1.0. Data are represented as the mean ± S.D. from three independent experiments (vs non-transduction *p < 0.05, **p < 0.01)

To identify the human brain endothelial fate-inducing factors, three TFs, Zic3A, Zic3B, and Sox18, which are involved in BMEC development, were selected and transfected to HUVECs using a lentivirus vector. Three days later, HUVECs transduced with Sox18 expressed a similar level of EC markers (PECAM1 and VE-cadherin) as in the control group while upregulating ABCB1 expression. Moreover, HUVECs transduced with Zic3A or Zic3B upregulated the expression of Plvap, which is expressed at a low level in BMECs. In contrast, HUVECs transduced with Sox18 showed a similar expression of Plvap to that of the control group (Fig. 1). These results suggested that Sox18 has the potential to induce BMEC properties.

**Fig.2:**
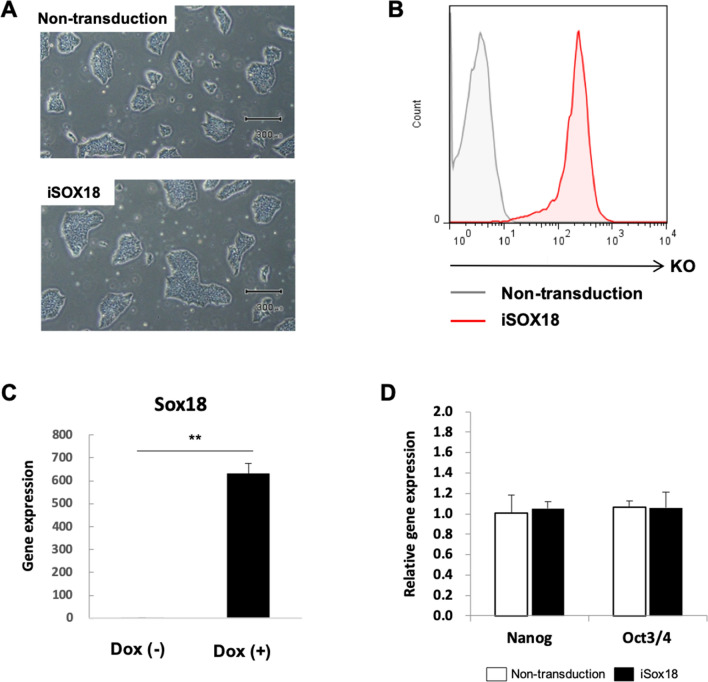
Dox-inducible Tet-on system induced SOX18 expression. **A** Phase-contrast micrographs of undifferentiation in Dox-inducible Sox18-iPS cells. One representative image from five independent experiments is shown. Scale bar = 300 μm. **B** Kusabira orange (KO) expression levels were analyzed using flow cytometry. Viable cells were gated to analyze the expression of surface markers using forward scatter versus side scatter (FSC vs. SSC). Representative data from one out of three independent experiments is shown. **C** Cells were treated with 0.1 ng/mL Dox, and the SOX18 expression levels was analyzed using qRT-PCR. Data are represented as the mean ± SD from three independent experiments (**p < 0.01). **D** Nanog and Oct3/4 expression level were analyzed using qRT-PCR. In the cells of the control group (non-transduction), the expression level of the gene was set to 1.0. Data are represented as the mean ± SD from three independent experiments

SOX18 expression was controlled using a Dox-inducible Tet-on system based on a reverse tetracycline-controlled transactivator (rtTA) and TRE. iSox18-iPSCs showed no difference in cellular morphology compared to non-transfected iPSCs (Fig. 2A), and Kusabira orange (KO)-positive iSox18-iPSCs indicated that the gene of interest was readily inserted into iPS cells (Fig. 2B). iSox18-iPSCs also maintained iPSC characteristics for expressing undifferentiated marker genes, such as Nanog and Oct-3/4 (Fig. 2C), while expressing SOX18 functionally with the Dox-inducible system (Fig. 2D).

### Sox18-mediated iBMECs exhibited tight barrier properties

**Fig.3:**
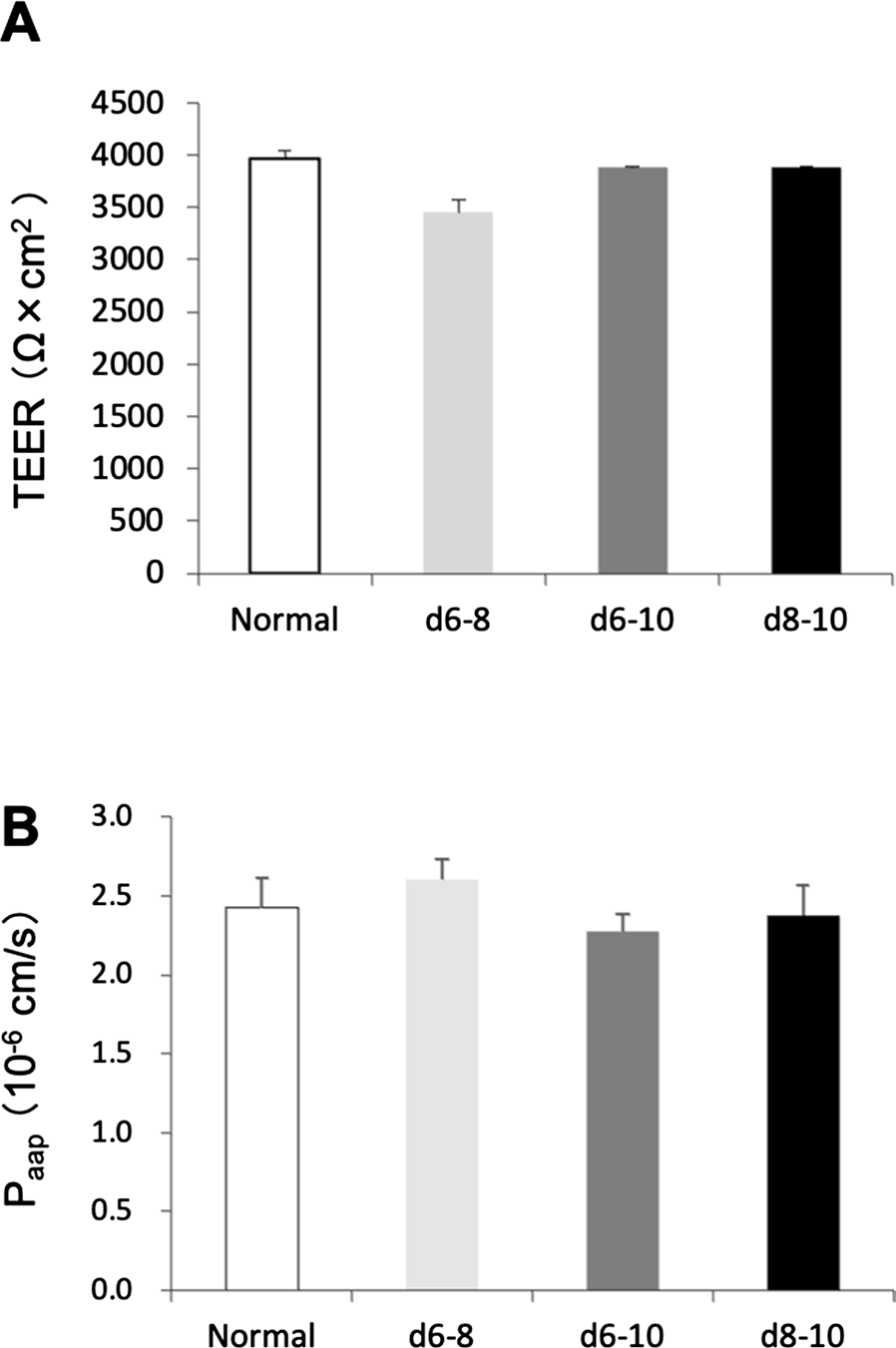
Evaluation of Sox18-mediated iBMEC trans-endothelial electrical resistance (TEER). **A** The effects of SOX18 expression on the TEER value on day 10 of iBMECs are shown. Data are presented as the mean ± SD of triplicate wells. **B** Sodium fluorescein permeability was measured. Data are presented as the mean ± SD of triplicate wells

It is known that gene silencing of lentiviral transduction often occurs in iPSC systems during differentiation. When we counted the number of KO-positive cells in iBMECs using flow cytometry on day 10, more than 95% of positive cells were detected (Additional file 1: Fig. S1). On day 10, we measured TEER and sodium fluorescein (NaF) permeability to evaluate barrier tightness and integrity. Sox18-mediated iBMECs achieved a high TEER value similar to normal iBMECs (> 3000 Ω cm^2^), suggesting that SOX18 expression did not interfere with the iBMECs barrier formation during differentiation (Fig. 3A). In addition, no significant differences in NaF permeability were found between Sox18-mediated iBMECs and normal iBMECs (Fig. 3B), consistent with the high TEER value of Sox18-mediated iBMECs.

**Fig.4:**
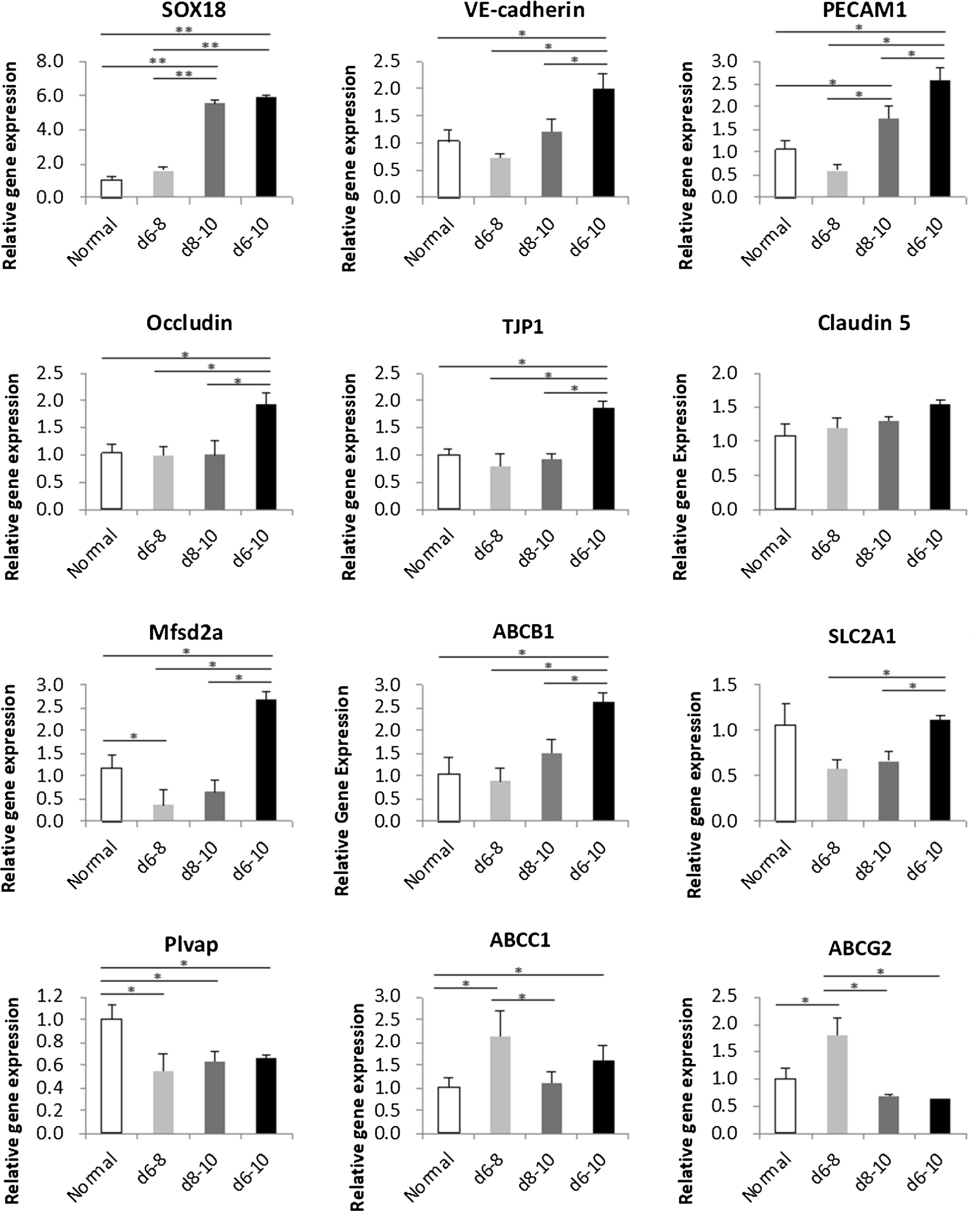
Expression of blood–brain-barrier (BBB) markers in Sox18-mediated iBMECs. qRT-PCR analysis of purified RNA from normal iBMECs, Sox18-mediated iBMECs (Dox d6–8), Sox18-mediated iBMECs (Dox d8–10), and Sox18-mediated iBMECs (Dox d6-10) on day 10. In normal iBMECs cells, RNA expression levels were set to 1.0. Data are presented as the mean ± S.D. of triplicate wells (*p < 0.05, **p < 0.01)

Using qRT-PCR, we confirmed the expression of multiple genes essential for BMEC development and function on day 10 of culture. Sox18-mediated iBMECs in the d8–10 and d6–10 groups showed significant Sox18 mRNA expression on day 10, whereas the d6–8 group expressed Sox18 mRNA at a similar level to normal iBMECs that were cultured in the absence of Dox, suggesting that SOX18 expression was regulated by the Dox-inducible system. EC marker (PECAM1 and VE-cadherin) expression in the d6–10 group was almost twice that in the normal groups (Fig. 4), consistent with the result of protein expression from immunocytochemistry (Additional file 1: Fig. S2).

Moreover, the d6–10 group expressed a series of tight junction markers (CLDN5, TJP1, and OCLN), which contributed to the barrier properties of the BBB, consistent with the evaluated high TEER value on day 10. The expression of Mfsd2a, a specific marker of the BBB, was significantly upregulated in the d6–10 group (Fig. 4). Plvap is known to be expressed in the BBB during development and is downregulated with the onset of barrier properties. Thus, the high expression of Mfsd2a and low expression of Plvap in the d6–10 group could correspond to the BBB specification and maturation. The d6–10 group also expressed SLC2A1/Glut1 at levels as high as in normal iBMECs, along with the upregulation of ABCB1/P-gp (Fig. 4A). We also compared the protein expression level of P-gp between the d6-10 group and the normal group. Our results showed that the P-gp protein level was increased in the d6-10 group (Additional file 1: Fig. S3). This result was consistent with the qRT-PCR results. These results indicate that SOX18 expression (treatment with Dox) from days 6 to 10 could induce the maturation of iBMECs.

**Fig. 5:**
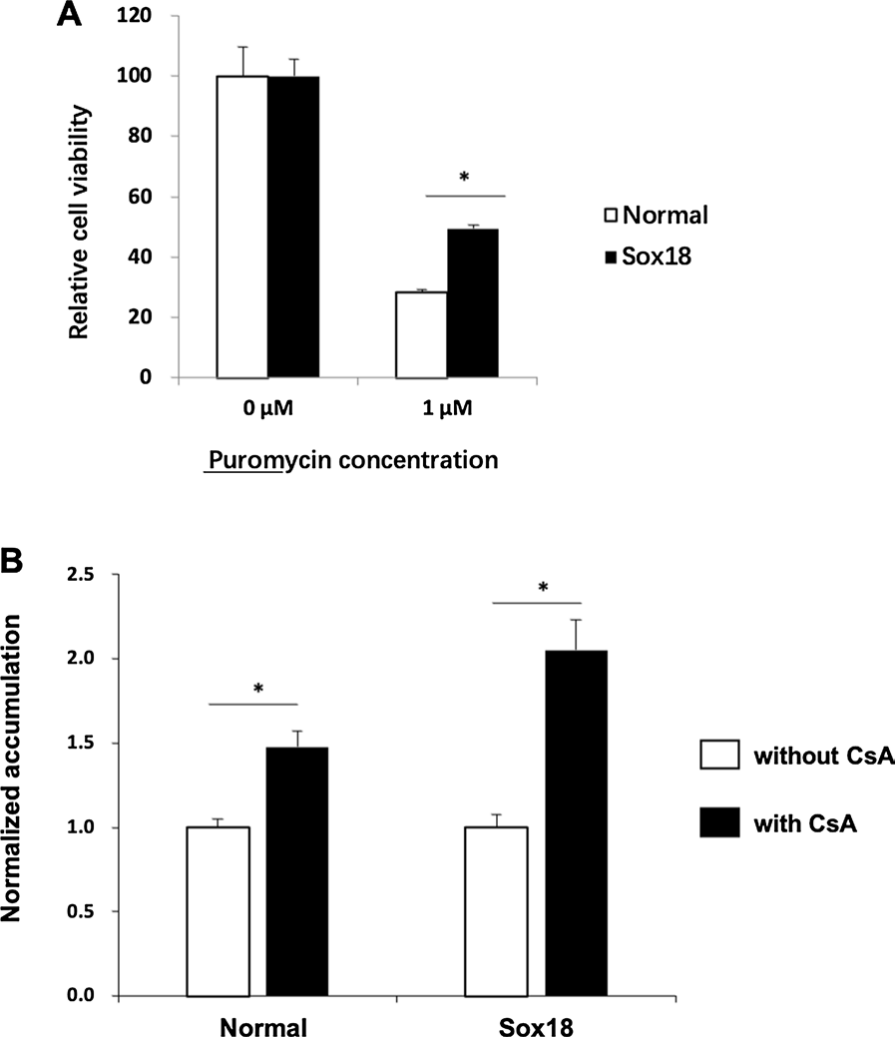
Sox18-mediated P-gp activity in iBMECs. **A** The relative viability of Sox18-mediated iBMECs (Dox d6-10) and normal iBMECs is shown. Cell viability without puromycin treatment (0 µM) was set to 100. Data are presented as the mean ± S.D. of triplicate wells (*p < 0.05). **B** iBMECs were incubated with rhodamine-123 (10 μM) in the absence or presence of CsA (10 μM). Relative fluorescence intensity values were normalized to each cell number and self-normalized to the conditions without inhibitor. Data are the mean ± S.D. from triplicate wells (*p < 0.05)

Puromycin, a substrate of P-gp, is toxic to prokaryotic and eukaryotic cells. We assumed that P-gp expression in iBMECs would transport puromycin to the outside of cells, resulting in higher cell viability. On day 10, Sox18-mediated iBMECs (Dox d6-10) were treated with puromycin. Two days later, cell viability was measured using the alamarBlue assay, a measure of mitochondrial fitness that provides a surrogate endpoint for the cell number. We found that Sox18-mediated iBMECs showed higher viability than normal iBMECs (Fig. 5A). Finally, we examined the potential effects of SOX18 on P-gp efflux transporter functionality via intracellular accumulation of the fluorescent P-gp substrate (rhodamine-123) in the presence and absence of the P-gp inhibitor (CsA). Both normal iBMECs and Sox18-mediated iBMECs exhibited an increase in rhodamine-123 accumulation in the presence of CsA (Fig. 5B). In addition, more significant accumulation of rhodamine 123 in the presence of CsA was observed in Sox18-mediated iBMECs (Fig. 5B). These results indicating that SOX18 expression could upregulate P-gp activity in iBMECs.

## Discussion

There have been reports about inducing BBB character in hPSC-derived BMEC-like cells with supplements of ATRA, insulin, VEGF, or other factors like hypoxia, shear stress, and three dimensional architecture [3, 24–26]. In this study, we explored the potential of driving BMEC phenotype in iBMECs with related TFs. We chose three TFs, Zic3A, Zic3B, and Sox18, and hypothesized that they are BBB phenotype-inducing TFs. To explore their effects on the induction of BBB phenotype, these TFs were transduced into HUVECs. The results showed that HUVECs transduced with Sox18 expressed P-gp and Plvap in a pattern similar to that of BMECs (Fig. 1), indicating the potential of Sox18 in BBB phenotype induction.

Sox18 encodes a member of the SOX family of developmental transcription factors. In mice, Sox18 is involved in embryonic and postnatal angiogenesis [12–14], and in zebrafish, the knockdown of Sox18 alters arteriovenous specification [15–17]. In our study, we used a Dox-inducible Tet-on system to induce SOX18 expression during iBMEC differentiation (Fig. 2). Compared to lentiviral transduction, the Dox-inducible Tet-on system is more suitable for gene expression modulation. The Dox-inducible Tet-on system is based on the bacterial Tet operon, which allows activation of gene expression by Dox and is widely used to control gene expression in mammalian cells [18]. We treated Dox-inducible Sox18-iPS cells with Dox to regulate SOX18 expression at specific differentiation stages.

We observed upregulation of tight junction protein expression in Sox18-mediated iBMECs (Fig. 4), consistent with previous reports that Sox18 is a strong EC barrier-inducing TF [7]. However, even though Sox18-mediated iBMECs reached high TEER values, they showed no increase compared with normal iBMECs TEER (Fig. 3). We speculated that this was because the barrier function of iBMECs was originally high, and the upregulation of tight junction protein expression was insufficient to increase TEER in Sox18-mediated iBMECs significantly.

We also found upregulation of EC markers (VE-cadherin and PECAM1) and BBB transporters (P-gp and Mfsd2a) in Sox18-mediated iBMECs. Sox18 is expressed transiently in the endothelial component of developing blood vessels in mice and chickens [15, 19] and is involved in canonical Wnt signaling [14]. Multiple studies have demonstrated that Wnt/β-catenin signaling promotes the BBB phenotype in BMEC cultures [20, 21]. We believe that Sox18-induced EC marker upregulation and BBB property acquisition are related to Wnt/β-catenin signaling activity, and further studies on Sox18-mediated iBMECs maturation mechanisms are essential. In addition, ABCC1 and ABCG2 expression was also upregulated in the d6–8 group, whereas the d8–10 and d6–10 groups expressed a similar level to normal iBMECs (Fig. 4). Given that ABCC1 and ABCG2 were found to be expressed in epithelial and endothelial cells [22, 23], there is a possibility that the d6–8 group included epithelial cells. However, more experiments are needed to explore the expression of epithelial markers in each group. Besides, the Sox18-mediated iBMECs maturing pattern need more investigation on other iPS cell lines to obtain the similar results.

## Conclusions

We used the Dox-inducible Tet-on system to regulate SOX18 expression during iBMEC differentiation. Inducible SOX18 expression in iBMECs resulted in BBB phenotypes, including high TEER value, upregulation of tight junction-related genes or protein, EC markers, and BBB transporters, and higher cell viability following treatment with puromycin. Collectively, we provide a differentiation method for the maturation of human iPS cell-derived BMECs with SOX18 expression, showing its contribution in forming an in vitro BBB model.

## Supplementary Information

.Additional information and additional Figure S1, S2, and S3.

## Data Availability

The datasets used and/or analyzed during the current study are available from the corresponding author on reasonable request.

## Abbreviations

ABCB1: ATP Binding Cassette Subfamily B Member 1
ABCC1: ATP Binding Cassette Subfamily C Member 1
ABCG2: ATP Binding Cassette Subfamily G Member 2
ATRA: All-trans retinoic acid
BBB: Blood-brain barrier
bFGF: Basic fibroblast growth factor
BMECs: Brain microvascular endothelial cells
CLDN5: Claudin-5
CNS: Central nervous system
Dox: Doxycycline
EC: Endothelial cell
ECM: Extracellular matrix
GAPDH: Glyceraldehyde 3-phosphate dehydrogenase
HUVECs: Human Umbilical Vein Endothelial Cells
iBMECs: BMECs derived from iPSC
iPSCs: Induced pluripotent stem cells
iSox18: Inducible Sox18
KO: Kusabira orange
Mfsd2a: Major Facilitator Superfamily Domain Containing 2A
MOI: Multiplicity of infection
mTeSR1: Modified Tenneille Serum Replacer 1
OCLN: Occludin; Oct3/4: Octamer-binding transcription factor
PDS: Platelet-poor plasma derived serum
PECAM1: Platelet endothelial cell adhesion molecule
P-gp: P-glycoprotein; Plvap: Plasmalemma Vesicle Associated Protein
rtTA: Reverse tetracycline-controlled transactivator
SLC2A1: Solute Carrier Family 2 Member 1
SOX: Sry-related high mobility group box
STAT3: Signal transducer and activator of transcription 3
TEER: Trans-endothelial electrical resistance
TF: Transcription factor
TJP1: Tight junction protein 1
TRE: Tetracycline-responsive element promoter
VE-cadherin: Vascular endothelial-cadherin
Zic: Zinc-finger protein of the cerebellum
