# Supplementary material for: The maturation of iPS cell-derived brain microvascular endothelial cells by inducible-SOX18 expression
Source: Fluids Barriers CNS. 2023 Feb 2;20:10. doi: 10.1186/s12987-023-00408-5 (PMC9893670; doi:10.1186/s12987-023-00408-5)
Supplement: Supplementary file 1 — .Additional information and additional Figure S1, S2, and S3. [file 12987_2023_408_MOESM1_ESM.docx]

**ADDITIONAL FILE 1**

The maturation of iPS cell-derived brain microvascular endothelial cells by inducible-SOX18 expression

Hongyan Zhang^1,2^, Tomoko Yamaguchi^2^, and Kenji Kawabata^1,2*^

^1^Laboratory of Biomedical Innovation, Graduate School of Pharmaceutical Sciences, Osaka University; 1-6 Yamadaoka, Suita, Osaka 565-0871, Japan;

^2^Laboratory of Cell Model for Drug Discovery, National Institutes of Biomedical Innovation, Health, and Nutrition, Saito-Asagi 7-6-8, Ibaraki, Osaka 567-0085, Japan.

*To whom correspondence should be addressed.

Kenji Kawabata, PhD; Laboratory of Cell Model for Drug Discovery, National Institutes of Biomedical Innovation, Health, and Nutrition, Saito-Asagi 7-6-8, Ibaraki, Osaka 567-0085, Japan

Phone: +81-72-641-9815

FAX: +81-72-641-9816

E-mail: kawabata@nibiohn.go.jp

**Additional Information**

*Immunocytochemistry*

Wash the cells with PBS at room temperature, and fix them using ice-cold methanol for 15 min. After PBS washing, the cells were incubated with Normal Goat Serum (10%, Wako) in PBS supplemented with 0.1% Triton-X for 1 h at Room Temperature. Then cells were stained with a rabbit anti-vascular endothelial-cadherin (VE-cadherin) antibody (diluted 1:50; Cell Signaling Technology) and rabbit anti-platelet endothelial cell adhesion molecular-1 (PECAM-1) antibody (diluted 1:50; Thermo Fisher Scientific) at 4°C overnight. Incubate the cells with Alexa Fluor 488-conjugated secondary antibodies (1:1000; Thermo Fisher Scientific) for 1 h at room temperature. The cells were washed and labeled with 4’,6-diamidino-2-phenylindol (DAPI) (Sigma). The green fluorescent images in the cells were monitored using a BZ-X710 microscope (KEYENCE).

*Western blot analysis*

The resulting cells were washed 3 times with sterile PBS and lysed in Radio-Immunoprecipitation assay buffer (RIPA buffer, Thermo Fisher Scientific) supplemented with Protease Inhibitor Cocktail Tablets (Roche). They were resolved by SDS-PAGE on a 5–20% polyacrylamide gel (Wako), and subjected to Western blot analysis as described previously (ref). P-gp was detected using 1/100 diluted a mouse anti-P-gp antibody (Gene Tex) and HRP-conjugated anti-mouse IgG (Cell Signaling Technology). The bands were detected with ECL Plus western blotting reagents (Thermo Fisher Scientific), and the signals were visualized with a LAS-4000 imaging system (Fuji Film).

**Additional Figure**


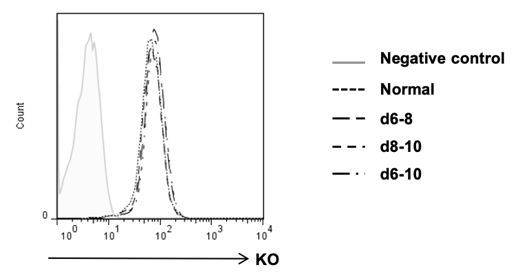


**Fig. S1. Expression of Kusabira orange (KO) in Sox18-mediated iBMECs**

Expression of KO in normal iBMECs, Sox18-mediated iBMECs (Dox d6-8), Sox18-mediated iBMECs (Dox d8-10), and Sox18-mediated iBMECs (Dox d6-10) were analyzed using flow cytometry. Viable cells were gated to analyze the expression of surface markers using forward scatter versus side scatter (FSC vs. SSC). Representative data from one out of three independent experiments is shown.


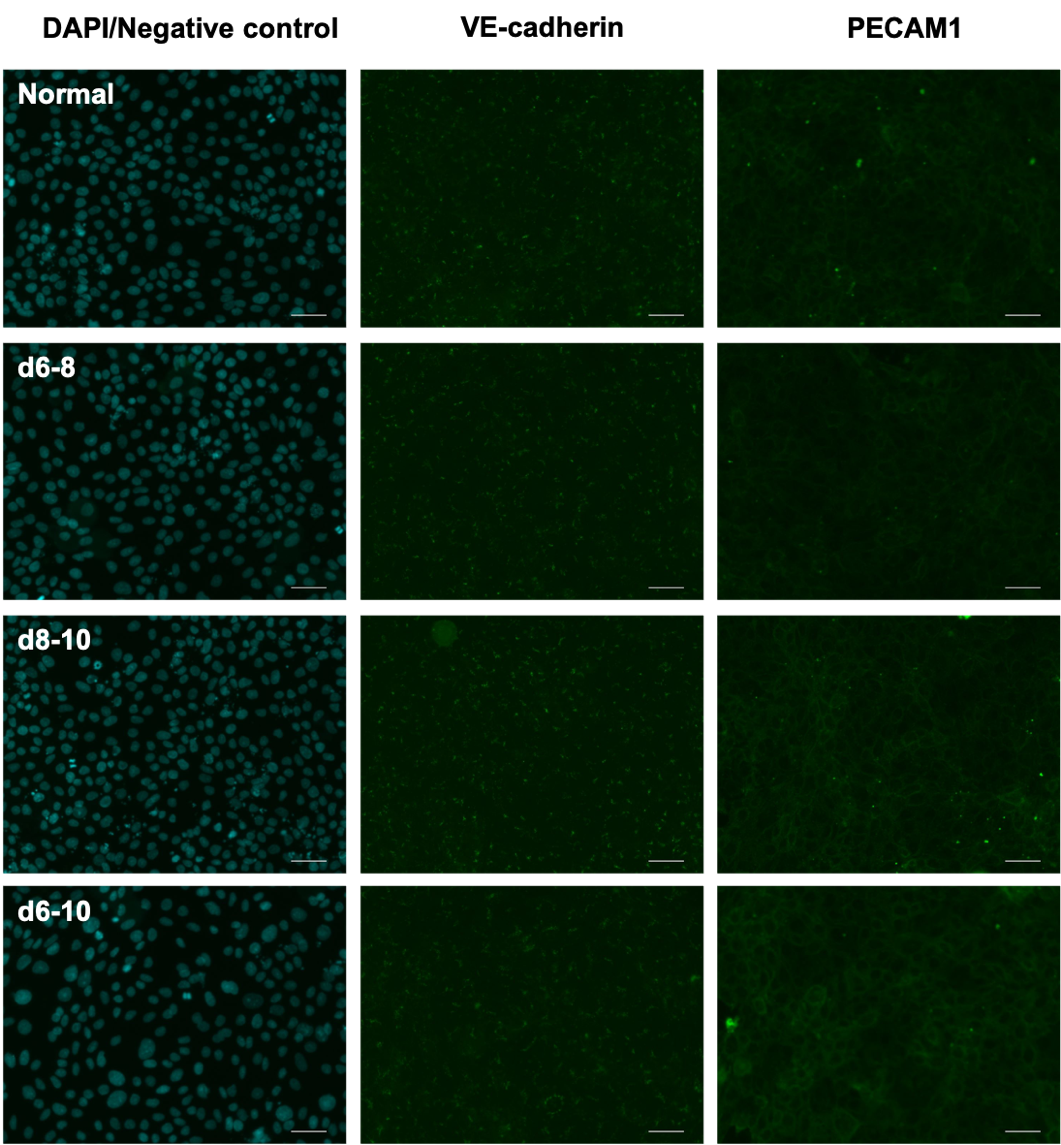


**Fig. S2. Expression of EC markers in Sox18-mediated iBMECs**

Expression of the VE-cadherin (green) or PECAM1 (green) in normal iBMECs, Sox18-mediated iBMECs (Dox d6-8), Sox18-mediated iBMECs (Dox d8-10), and Sox18-mediated iBMECs (Dox d6-10) were evaluated by immunocytochemistry. One representative image from three independent experiments is shown. Scale bar = 50 µm.


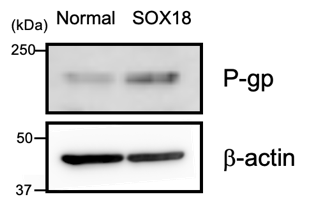


**Fig. S3. Expression of P-gp protein in Sox18-mediated iBMECs**

Expression protein levels of P-gp in normal iBMECs and Sox18-mediated iBMECs (Dox d6-10) were evaluated by western blotting. One representative image from three independent experiments is shown.
